# Repurposing the anti-malarial drug dihydroartemisinin suppresses metastasis of non-small-cell lung cancer via inhibiting NF-κB/GLUT1 axis

**DOI:** 10.18632/oncotarget.13536

**Published:** 2016-11-24

**Authors:** Jie Jiang, Guojun Geng, Xiuyi Yu, Hongming Liu, Jing Gao, Hanxiang An, Chengfu Cai, Ning Li, Dongyan Shen, Xiaoqiang Wu, Lisheng Zheng, Yanjun Mi, Shuyu Yang

**Affiliations:** ^1^ Department of Thoracic Surgery, Xiamen Cancer Hospital, The First Affiliated Hospital of Xiamen University, Xiamen, People's Republic of China; ^2^ Department of Medical Oncology, Xiamen Cancer Hospital, The First Affiliated Hospital of Xiamen University, Xiamen, People's Republic of China; ^3^ Biobank, The First Affiliated Hospital of Xiamen University, Xiamen, People's Republic of China; ^4^ Sun Yat-sen University Cancer Center, State Key Laboratory of Oncology in South China, Collaborative Innovation Center of Cancer Medicine, Guangzhou, People's Republic of China; ^5^ Xiamen Diabetes Institution, The First Affiliated Hospital of Xiamen University, Xiamen, People's Republic of China

**Keywords:** dihydroartemisinin, non-small-cell lung cancer, Warburg effect, metastasis, NF-κB

## Abstract

Non-small-cell lung cancer (NSCLC) is an aggressive malignancy and long-term survival remains unsatisfactory for patients with metastatic and recurrent disease. Repurposing the anti-malarial drug dihydroartemisinin (DHA) has been proved to possess potent antitumor effect on various cancers. However, the effects of DHA in preventing the invasion of NSCLC cells have not been studied. In the present study, we determined the inhibitory effects of DHA on invasion and migration and the possible mechanisms involved using A549 and H1975 cells. DHA inhibited *in vitro* migration and invasion of NSCLC cells even in low concentration with little cytotoxicity. Additionally, low concentration DHA also inhibited Warburg effect in NSCLC cells. Mechanically, DHA negatively regulates NF-κB signaling to inhibit the GLUT1 translocation. Blocking the NF-κB signaling largely abolishes the inhibitory effects of DHA on the translocation of GLUT1 to the plasma membrane and the Warburg effect. Furthermore, GLUT1 knockdown significantly decreased the inhibition of invasion, and migration by DHA. Our results suggested that DHA can inhibit metastasis of NSCLC by targeting glucose metabolism via inhibiting NF-κB signaling pathway and DHA may deserve further investigation in NSCLC treatment.

## INTRODUCTION

Lung cancer is not only the most common malignant cancer but also the main cause of mortality by cancer globally. Moreover, there are almost 80% of cancer mortality in lung cancer occurred in one of predominant histological subtypes, non-small-cell lung cancer (NSCLC) [1–3]. Although a variety of therapies have been considerably improved in the past few years, invasion and metastasis still enormously limit treatment options, with no available cure for patients with advanced disease [4–7]. Therefore, inhibition of tumor metastasis is important in clinical practice.

DHA is likewise the active metabolite of all artemisinin compounds (artemisinin, artesunate, artemether, etc.) and nearly about 5 times more potent than artemisinin against malaria, Plasmodium falciparum [8–10]. Previous studies with DHA focused on its antimalarial abilities, while gradually its effective anticancer activity has been recognized as well. Previous researches have reported that DHA has been discovered function in inhibition of cell proliferation and induction of apoptosis by downregulating cyclin D1, Bcl-2, Bcl-xL, caspase, and VEGF, while upregulating P21, P27 and Bax in a series of cancer cell lines could be effected [11–14]. DHA was also applied to breast cancer-induced mouse osteolysis mode, that DHA can inhibit breast tumor-induced osteolysis via restraining the invasion and migration by regulating AKT signaling pathway [15]. Similarly, our recent studies indicated that DHA restrain glucose uptake. Furthermore, it can induce apoptosis by cooperation with glycolysis inhibitor in NSCLC cells [16]. Interestingly, a recent popular study reported that DHA reduces both the incidence and the risk of metastasis in osteosarcoma and colon cancer [17–19]. Even though DHA has been confirmed wide usefulness in treatment for many diseases, there is no evidence at the cellular level or in animal models for such an effect of DHA on NSCLC metastasis [20–22]. Moreover, a distinguished feature of various tumor cells is elevated aerobic glycolysis and increased glucose uptake. This metabolic reprogramming, known as the Warburg effect, provides an advantage for growing of tumor cells even in regions with hypoxia. Metastasis of cancer cells regulates by changes of glucose metabolism through high glucose uptake, high lactate formation, low extracellular pH and reduction of intratumoral pH [23–26].

In this paper, we discovered DHA inhibited migration and invasion of NSCLC cells even in low concentration with little cytotoxicity, and also inhibited Warburg effect. Mechanically, DHA negatively regulates NF-κB signaling to inhibit the GLUT1 translocation. Blocking the NF-κB signaling primarily abolishes the inhibitory influences of DHA on GLUT1 translocation to the plasma membrane and metastasis. GLUT1 knockdown significantly decreased migration and the inhibition of invasion by DHA.

## RESULTS

### DHA depresses the Warburg effect, migration and invasion in NSCLC cells

**Figure 1:**
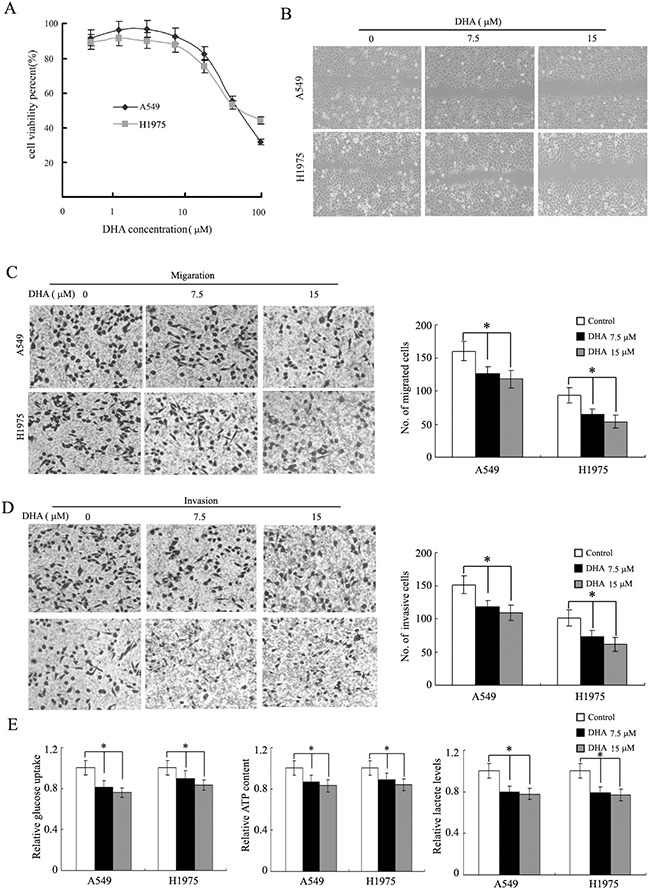
DHA depresses the Warburg effect, migration and invasion of NSCLC **A**., DHA inhibits NSCLC cell (A540 and H1975) viability in dose-dependent manner. **B**., DHA inhibits cell migration in the wound-healing assay. Shown here are representative images of three independent experiments. **C** and **D**., DHA inhibits A549 and H1975 cells migration and invasion in a Transwell assay. The representative photographs of migration and invasion cells were shown as C and D, respectively. **E**., DHA decreases glucose uptake, cell ATP content and lactate levels in A549 and H1975 cells. Columns, mean of three determinations; bars, SD. *, P < 0.05; **, P < 0.01, control versus DHA-treated cells.

First of all, the dose of dependence of cytotoxic effects of DHA in A549 and H1975 cells was determined by using the MTT assay As shown in Figure 1A and Supplementary Figure S1. Then the pharmacological potential of DHA on antimetastasis activity was investigated. It was found that DHA reduced cell viability in a dose-dependent manner. DHA at concentration lower than 15μM had no apparent cytotoxic act on the cells and DHA at 30μM showed about a 15-20% decrease in cell viability. We next used the wound-healing assay and transwell assay to investigate the migratory abilities of the two NSCLC cells treated with 7.5μM and 15 μM DHA. Treatment with DHA for 24h reduced the migratory abilities of these lung cancer cells in a dose-dependent manner (Figure 1B). Subsequently, the effects of DHA on the invasion of these cells were also determined by matrigel invasion assay system. At 7.5 and 15 μM DHA, the invasion capability cells were inhibited 20.7% and 26.5% in A549 cell, 25.1% and 31.7% in H1975 cells respectively, compared to the control group (Figure 1C and 1D). We found that 7.5 and 15 μM DHA decreased ATP production, glucose uptake and lactate levels in NSCLC cells (Figure 1E).

### DHA inhibits the metastasis of NSCLC *in vivo*

**Figure 2:**
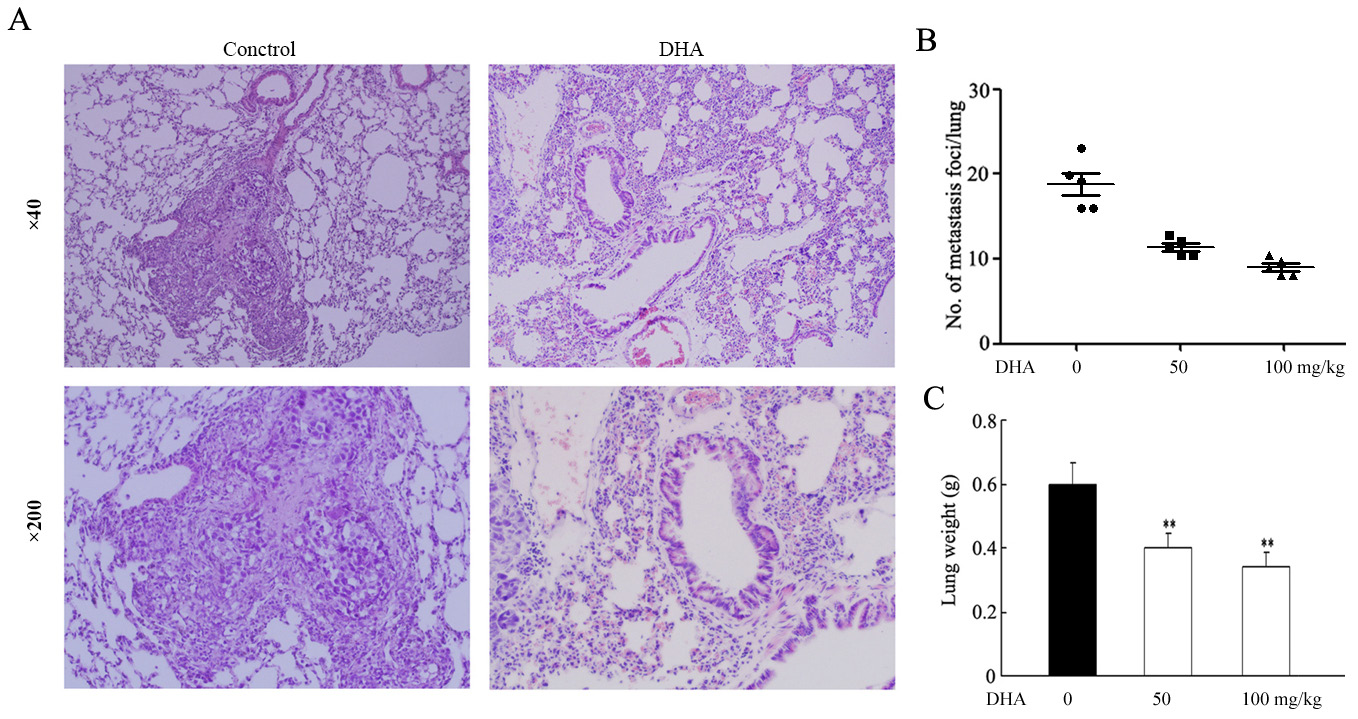
Effect of DHA on the metastasis of A549 in nude mice Following the experimental design as described in Materials and Methods, nude mice were sacrificed at day 28 to evaluate lung metastases. **A**., the image is a representative H&E-stained section of lung metastases. **B**., the number of metastatic nodules in the lungs of nude mice using a Mann–Whitney test. Dots, scores. Bars, SD; *, P < 0.05. C., the wet lung weights from the tumor bearing nude mice. Bars, SD; *, P < 0.05. A two-tailed Student t test was used for statistical analysis.

To determine whether DHA treatment could reduce metastasis *in vivo*, A549 cells were directly injected into the tail vein of female nude mice. After injection, mice received DHA treatment for 4 weeks. We found that the formation of metastases in the lung was reduced by 45.3% for the C1 group (50mg/kg/d), and 56.2% for the C2 group (100mg/kg/d) (Figure 2A and 2B). Additional evidence of the inhibition of metastasis in the DHA-treated group was confirmed by the significant difference in whole-lung wet weights among control and DHA treatment groups (Figure 2C). DHA treatment was well tolerated, as determined by stable body weights throughout the 28 days’ treatment period (data not shown). In general, these data suggest that DHA inhibited metastasis of NSCLC cells *in vivo*.

### DHA inhibits transcriptional activity of the NF-κB gene

**Figure 3:**
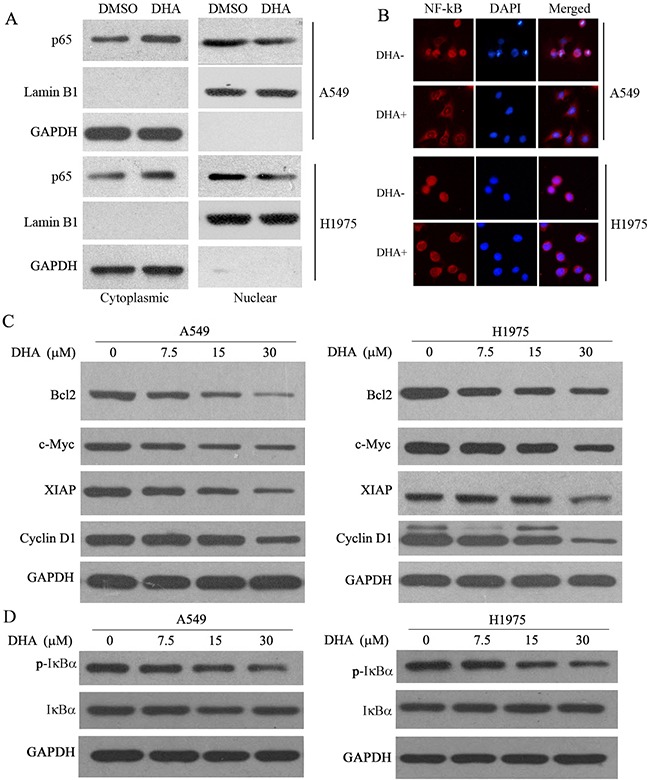
DHA inhibits the NF-κB pathway **A**. Cytoplasmic and nuclear extracts from these cells were immunoblotted for p65, Lamin B1 (nucear marker) and GAPDH (cytoplasmic marker GAPDH). **B**., Immunocytochemistry showing the effect of DHA inhibited p65 nuclear translocation in A549 and H1975 cells. Inhibition of p65 nuclear translocation by DHA. The indicated cells were treated with or without DHA (15 μM) for 48 hours and then subjected to cell fractionation and analyzed by Western blotting. **C**., downstream targets of NF-κB, such as BCL2, c-Myc, and XIAP, were downregulated by DHA in a dose-dependent manner in both A549 and H1975 cells. The indicated cells were treated with different concentrations of DHA for the indicated times and then analyzed by Western blot analysis. Actin was used as a loading control; n=3. **D**., DHA inhibites IKK activation and IkB degradation in A549 and H1975 cells. The indicated cells were treated with different concentrations of DHA and then analyzed by Western blot analysis. GAPDH was used as a loading control. Cell lysates were immunoblotted for phospho- and total IkB.

It has been proclaimed that inhibition of NF-κB signaling pathway plays a critical role in regulating gene expression [27, 28]. A549 and H1975 cells were transfected with NF-κB luciferase plasmid, and then incubated with DHA (15 μM) for 24 hours in order to investigate the effect of DHA on the transcriptional activity. The NF-κB luciferase reporter activity was decreased by 30% after treated with DHA in NSCLC cells (Supplementary Figure S2). Consistently, the nuclear localization of p65 protein, the indicator of NF-κB transcription activity, was also decreased in NSCLC cells by DHA treatment (Figure 3A). These results were conformed by immunostaining of p65 in A549 and H1975 cells (Figure 3B), indicating that DHA can inhibition NF-κB nuclear translocation. In addition, the NF-κB target genes, including cyclinD1, XIAP, BCL2 and c-myc were down-regulated by DHA (Figure 3C). These results together indicated that DHA inhibits the NF-κB signaling.

### DHA negatively regulates the migration and invasion of NSCLC through down-regulation of the NF-κB signaling

**Figure 4:**
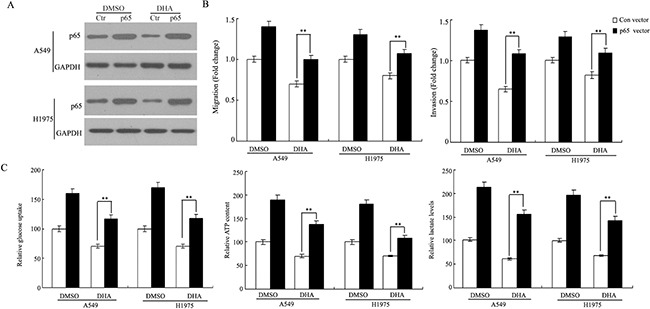
DHA negatively regulates the Warburg effect, migration and invasion of NSCLC through down-regulation of the NF-κB signaling A549 and H1975 cells were transfected with control (Ctr) and p65 vector for 8 h respectively, and then treated with the indicated concentration of DHA for 48 h. **A**. Cell lysates were subjected to western blot analysis with the indicated antibodies. **B**. Ectopic expression of p65 largely abolished the inhibitory effect of DHA on A549 and H1975cells migration and invasion in a Transwell assay. Cells transfected with p65 and control cells were treated with indicated concentrations of DHA for 48h before assays. **C**. Ectopic expression of p65 largely abolished the inhibitory effect of DHA on glucose uptake, cell ATP content and lactate production in A549 and H1975 cells. This experiment was repeated thrice. Columns, mean; bars, SD. ***, P < 0.05.

The NF-κB activation has been reported to improve the Warburg effect, migration and invasion in cancer cells [29–31]. Here, we explored whether the inhibition of DHA on the Warburg effect, migration and invasion depends on the NF-κB signaling in lung cancer cells. As shown in Supplementary Figure S3, DHA had no effect on the Warburg effect, migration and invasion of NSCLC cells knockdown of p65 by siRNA in these cells (P > 0.05). Conversely, as shown in Figure 4A-4C, the impairment of DHA on these events were rescued by in these NSCLC cells overexpressing p65. These results suggested that DHA negatively regulates the Warburg effect, migration and invasion through its down-regulation of the NF-κB signaling.

### DHA inhibits plasma membrane location of Glut 1

**Figure 5:**
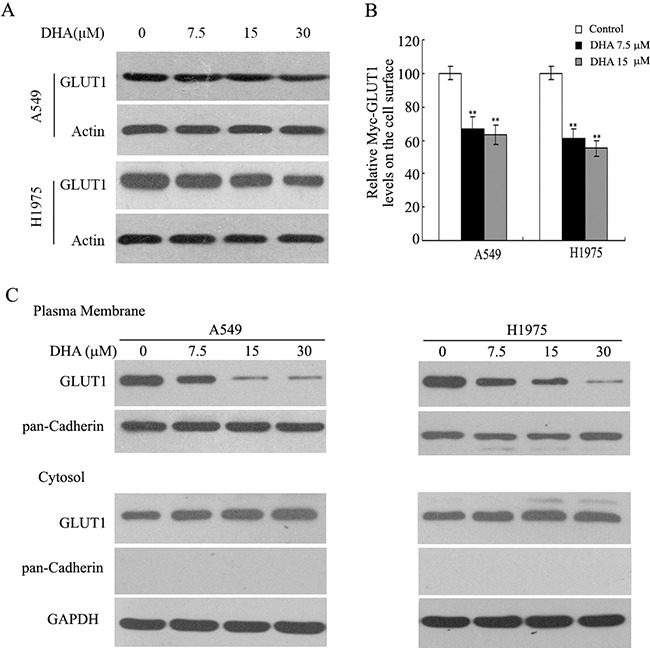
Inhibition of GLUT1 membrane localization by DHA **A**. A549 and H1975 cells were treated with either DMSO (control) or DHA for 24 h. GLUT1 levels were evaluated by immunoblot analysis, and actin was used as the loading control. **B**. Inhibition of GLUT1 membrane trafficking by DHA in NSCLC cells. At 48 h after transduction,GLUT1 membrane localization was verified in response to DHA by flow cytometry analysis the levels of Myc-GLUT1 on cell surface were analyzed in a flow cytometer and normalized with the total Myc-GLUT1 levels in cells. **C**. Membrane bounded GLUT1 inhibited by DHA in NSCLC cells. A549 and H1975 cells were incubated with DHA or DMSO containing media for 24 h and Western blotting was performed with protein, isolated from the plasma membrane or the cytosol.

To understand the mechanism of the dysregulation of Warburg effect by DHA, protein expression of glucose transporters GLUT1 in NSCLC cells was tested. As shown in Figure 5A, DHA did not inhibit the protein expression of GLUT1 in A549 and H1975 cells. Furthermore, myc-GLUT1 vectors that express GLUT1 with Myc tag in its first exofacial loop transduced cells, and the level of it on the cell surface were measured by immunofluorescence (IF) staining with an anti-Myc antibody followed by flow cytometry analysis. As shown in Figure 5B, DHA inhibited the level of Myc-GLUT1 on cell surface, compared with the control group in both NSCLC cells. These results indicate that GLUT1 plasma membrane localization shown a tread of reduction in both cancer cells treated with DHA. Plasma membrane protein was isolated for the evaluation of GLUT1 trafficking to confirm the dysregulation of GLUT1 plasma membrane trafficking in NSCLC cells. DHA treatment reduced membrane bound GLUT1 expression, a response consistent with our Flow cytometric results (Figure 5C). These data suggest that DHA inhibits translocation of GLUT1 to cytoplasmic membrane in these NSCLC cells.

### Regulation of NF-κB to GLUT1 membrane localization in NSCLC cells

**Figure 6:**
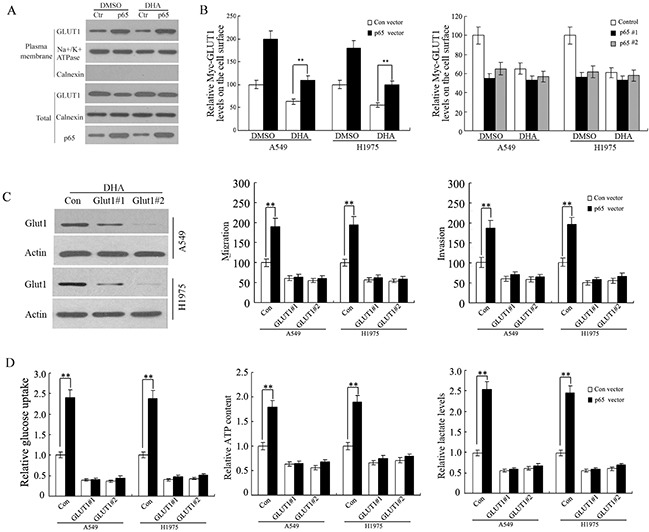
DHA inhibits GLUT1 translocation to the plasma membrane through down-regulating NF-κB signaling **A**. The NF-κB signaling promoted the translocation of endogenous GLUT1 to the plasma membrane in A549 and H1975 cells detected by westernblot assay. Cells were treated with DHA or DMSO (control) containing media for 48 h. **B**. Ectopic expression of p65 promoted the translocation of Myc-GLUT1 to the plasma membrane, and largely abolished the inhibitory effects of DHA on Myc-GLUT1 translocation to cell surface in cells measured by flow cytometry(left panel). P65 knockdown inhibited the translocation of Myc-GLUT1 to the plasma membrane (right panel) in A549 and H1975 cells. Cells were transfected with p65 expression vectors or p65-siR together with Myc-GLUT1 vectors. Cells were treated with DHA or DMSO (control) containing media for 48 h. The levels of Myc-GLUT1 on cell surface were analyzed in a flow cytometer and normalized with the total Myc-GLUT1 levels in cells. **C**. GLUT1 knockdown abolished the promoting effects of p65 on A549 and H1975cells migration and invasion. Cells were pre-transfected with 2 different GLUT1 siRNAs (GLUT1-siR) before transfection with p65 expression vectors. Left panel: Western-blot analysis of knockdown of GLUT1 by siRNA in cells. Left panel: Western-blot analysis of knockdown of GLUT1 by siRNA in cells. **D**. GLUT1 knockdown greatly abolished the promoting effects of p65 on glucose uptake, cell ATP content and lactate levels in A549 and H1975 cells. Cells were pre-transfected with 2 different GLUT1 siRNAs (GLUT1-siR) before transfection with p65 expression vectors. Data are presented as mean value ± SD (n=3). *p < 0.05; **p < 0.01 (student's t test).

Previous studies have reported that the activated NF-κB signaling promotes the translocation of GLUT1 to the plasma membrane, which could be an important mechanism by NF-κB activating the Warburg effect. To determine whether Glut 1 translocation in response to DHA in A549 and H1975 cells is dependent on the activation of NF-κB signaling pathway, Glut1 translocation was measured in transfected cells with p65 expression vectors or p65-siR with Myc-GLUT1 vectors. Ectopic expression of p65 greatly furthered the translocation of endogenous GLUT1 to the plasma membrane as shown by western-blot using the isolated plsama membrane fraction of A549 and H1975 cells (Figure 6A). Flow cytometric analysis of cell surface GLUT1 expression showed ectopic expression of p65 facilitated the translocation of Myc-GLUT1 to the plasma membrane. In addition, p65 knockdown inhibited the translocation of Myc-GLUT1 to the plasma membrane in A549 and H1975 cells (Figure 6B). These data start a raising a possibility that DHA inhibit GLUT1 translocation through its negative regulation of the NF-κB signaling. Consistent with this presume, our results clearly showed that activating the NF-κB signaling by ectopic expression of p65 mainly abolished the inhibitory effects of DHA on GLUT1 translocation to cell surface in A549 and H1975 cells (Figure 6A). Therefore, these findings suggest that DHA modulates the GLUT1 translocation to cell surface through down-regulating NF-κB signaling.

Furthermore, A549 and H1975 cells were pre-treated with 2 different GLUT1 siRNAs (GLUT1-siR) before transfection with p65 expression vectors. The invasion and migration were increased in p65-transfected cells, whereas GLUT1 knockdown greatly attenuated the promoting effects of p65 on in A549 and H1975 cells (Figure 6C). Moreover, knockdown of the endogenous GLUT1 by siRNA chiefly abolished the promoting effects of NF-κB activation on migration, invasion, glucose uptake, cell ATP content and lactate levels in A549 and H1975 cells with overexpression of p65 (Figure 6B and 6C). These results together indicate that DHA suppresses metastasis of non-small-cell lung cancer via inhibiting NF-κB/GLUT1 axis.

## DISCUSSION

Poor prognosis generally is discovered in NSCLC patients with metastatic or relapsed NSCLC. Thus, new improvement of therapies is needed for better treatment outcomes of NSCLC patients. As an alternative means, some pharmaceutical companies have adopted a drug repurposing to accelerate the drug discovery and development process. Drug repurposing, which is to identify novel and useful indications for existing drugs by targeting alternative diseases, has been proven to effectively locate unmet medical needs for cancer [32–35]. Drug repositioning in the United States is better established and this means to produce six drugs approved by the U.S. Food and Drug Administration (FDA) in 2009. New indications have been introduced by drug repositioning include aspirin, an NSAID, for colorectal cancer [36]; thalidomide, a sedative hypnotic agent, for multiple myeloma and leprosy [37]; a COX-2 inhibitor for pancreatic cancer and colorectal cancer [38, 39] and metformin, an antidiabetic drug, for endometrial cancer [40].

DHA possess anti-malarial activity and anti-cancer activities. As a new class of anticancer drugs, DHA have many advantages including low toxicity to normal tissue/cells [41], low cross-drug resistance [42], and synergistic effects with many traditional chemotherapeutic anticancer drugs [43]. The pharmacokinetics of DHA has been discussed in a few studies. DHA pharmacokinetics was not discovered prominent sexual differences in previous research that healthy volunteers taken oral DHA or AS in Vietnam [44]. The t1/2 of IP-administered DHA is approximately 40–70 min, whereas t1/2 of oral-administered DHA is predicted to be in the range of 0.8-1.5 hours. Oral DHA could be rapidly absorbed in the gastrointestinal tract; Cmax was acquired at approximately 1-2 hours of dosing. The pharmacokinetic profile can be seen as a one-compartment open model including first- order input and output, describing the rapid absorption, distribution and elimination phase [45]. In fact, Artesunate, another anti-malarial drug, has shown promise in crossover usage as an anti-cancer agent for a variety of solid tumors [46, 47]. A case study demonstrated a decrease of disease progression in a pituitary macroadenoma patient who treated with artemether for 12 months [48]. In another research of a randomized controlled trial investigated in 120 advanced non-small cell lung cancer patients, control group with 60 patients was treated with standard chemotherapy (vinorelbine and cisplatin) and another group with 60 patients was treated with 120 mg artesunate together with standard chemotherapy intravenously. The negligible side effects and improvement of short-term and one-year survival rates were detected in the group with the artesunate-treated patients, compared with control group [49]. DHA has recently been reported to reduce the incidence and risk of metastasis in breast cancer and osteosarcoma patients [50, 51]. However, the therapeutic efficacy and mode of action of DHA in NSCLC are not well understood. In this study, we demonstrated the efficiency of DHA on Warburg effect and metastasis of NSCLC *in vitro* and *in vivo*. In addition, our results showed that DHA diminished Warburg effect and metastasis of NSCLC cells probably via suppressing NF-κB/GLUT1 axis. The results may open a new avenue for the invention of novel agent in NSCLC therapy.

It has been reported that DHA suppressed cell migration and invasion in various types of tumor cells. Consistently, our results revealed that treatment of A549 and H1975 cells with low concentration of DHA had minimal impact on growth but strongly reduced migration and potential invasion, suggesting a role of DHA on cell motility and invasion without affecting proliferation. More important, our results provided evidences to indicate that DHA could reverse the Warburg effect in NSCLC cells. Moreover, therapy of targeting the pathway of glycolytic may be a new implication via killing the malignant cells because of association between increased metabolic activity and glucose concentration and aggressiveness of cancer. In this research, DHA inhibits the glucose uptake, the rate of glycolysis, and lactate production of NSCLC cells. These results might contribute to the consideration that DHA could restrain the metastasis and invasion of NSCLC cells as more aggressive tumors are seemly to have a greater need for glucose, frequently show this metabolic alteration. The increased glycolysis results in chronic acidification of the local environment through the conversion from pyruvate to lactic acid. This micro environmental acidosis leads to cancer cells invasion and metastasis.

The NF-κB signaling pathway is frequently activated in various types of human cancers, and thus agents capable of suppressing NF-κB activation may provide therapeutic prospect [5, 52–54]. Our results showed that NF-κB luciferase activity and NF-κB protein expression were inhibited by DHA, as well as NF-κB target genes, while ectopic expression of p65 largely abolished the inhibitory effect of DHA on NSCLC cell motility. These results are compliance with previous reports that DHA inhibited activation of NF-κB in other cancer cell lines and that resveratrol genistein, curcumin and aspirin, all present within DHA, were associated with NF-κB inactivation [55–59].

Studies reported that the NF-κB activation facilitates the Warburg effect in cancer cells. Our data also confirmed that the activation of NF-κB furthered the translocation of GLUT1 to the plasma membrane in NSCLC cells. DHA inhibited the GLUT1 translocation mainly through the down-regulation of NF-κB activities. Activating the NF-κB signaling mostly abolished the inhibitory effects of DHA on GLUT1 translocation and the Warburg effect. Furthermore, GLUT1 knockdown greatly attenuated the improving effects of p65 on glucose uptake, ATP content and lactate levels in A549 and H1975 cells. (Figure 6C). Meanwhile, GLUT1 knockdown significantly decreased the inhibition of invasion, and migration of lung cancer cells. These data indicated that DHA influenced the invasion and migration of lung cancer cells by regulating the expression of NF-κB, which changed the GLUT1 translocation.

Warburg effect has been addressed to use positron emission tomography (PET) with the glucose analog tracer in clinical oncology. The involvement of a series of onco-proteins and tumor suppressors, including p53, hypoxia-inducible factor, Myc and the PI3K/Akt/mTOR signaling pathway in the regulation of this metabolic adaptation that favors cellular proliferation, tumor growth and angiogenesis has been reported. Our previous studies have shown that DHA-suppressed glycolytic metabolism has connection with inhibition of mTOR activation [16]. Furthermore, the reduction of glucose uptake and ATP content was inhibited by overexpression of Rheb suppressed in DHA-treated A549 cells. In this study, we likewise found DHA inhibition on NF-κB signaling could be reverted by overexpression of Rheb in lung cancer cells (Supplementary Figure S4). It has been presented that inhibition of mTOR with rapamycin could prevent glucose uptake [60]. Additionally, a previous report has reported that mTOR/NF-κB pathway regulates aerobic glycolysis in vascular smooth muscle cells [61]. Therefore, this provided a possibility, GLUT1 expression may be decreased by DHA via mTOR/NF-κB pathway thereby inhibited glucose uptake.

In summary, the results from this study demonstrated that DHA could be a useful agent for inhibiting tumor metastasis in NSCLC. Also, GLUT1 modulation via NF-κB inactivation could be a critical cancer targeted strategy. On the basis of our findings, further in-depth researches including clinical trials are required to fully understand the value of DHA as an anticancer agent in NSCLC.

## MATERIALS AND METHODS

### Reagents and cell culture

A549 and H1975 cell lines were obtained from the American Type Culture Collection (ATCC) and maintained in DMEM (Gibco, Life Technologies) supplemented with 10% (v/v) fetal bovine serum (FBS) (Gibco, Life Technologies), 100 U/ml penicillin and 100 μg/ml streptomycin. Cells were grown in a humidified incubator at 37°C with 5% CO_2_. Cells were grown in monolayer and passaged routinely 2–3 times a week.

Antibodies against GLUT1 were purchased from Abcam (Cambridge, UK); The specific primary antibodies for p65, actin, Pan-Cadherin, cyclin D1, c-myc, XIAP, and lamin B1 were purchased from Cell Signaling Technology. Rabbit polyclonal antibodies specific for BCL-2 was purchased from Assay Biotechnology (Sunnyvale, CA, USA). An antibody against glyceraldehyde-3-phosphate dehydrogenase (GAPDH) antibodies were purchased from Kangchen Co. (Shanghai China). DHA was purchased from Sigma-Aldrich. For drug treatment, DHA were dissolved in DMSO; aliquots were stored at -80°C. Stock solutions were diluted to the desired final concentrations with growth medium just before use. Cells were seeded in triplicate at a density of 0.1–0.2 million/well in six well plates. Prior to drug treatment, cells were incubated for at least 12 h and thereafter replaced with media containing drugs, followed by 24 or 48 h incubation. DMSO-treated cells were used as a mock control.

### Cell viability

The MTT assay was performed as previously described to determine the sensitivity of cells to drugs [62]. Briefly, cells (3 × 10^3^ per well) were plated in 96-well plates in DMEM supplemented with 10% FBS and incubated at 37°C with 5% CO2. After 24 h of incubation, DHA at various concentrations was added into the wells, followed by additional incubation for 68 h. MTT was then added to the wells and cells were incubated for additional 4 h. Finally, the optical density at 490 nm was measured using a multi-well plate reader (Micro-plate Reader, Bio-Rad). The 50% inhibitory concentration (IC50) was determined as the anticancer drug concentration causing 50% reduction of cell viability and calculated from the cytotoxicity curves (Bliss method) [32]. Cell survival was calculated using the following formula: survival (%) = (mean experimental absorbance/mean control absorbance) × 100%.

### Measurements of glucose levels and lactate production of cells in the media

Per well of a 12-well plate was seeded with a total of 3×10^5^cells treated with various drugs. For assessment of glucose uptake, the media were collected and the glucose was immediately measured using an Olympus AU5400 (Olympus Corporation, Tokyo, Japan). For assessment of lactate production, the media was collected and diluted 1:100 in lactate assay buffer. The amount of lactate present in the media was then estimated using the Lactate Assay Kit (sigma, St. Louis, MO, USA) according to the manufacturer's instructions.

### ATP assay

Cell ATP content was determined over time using the ATP Bioluminescent Somatic Cell Assay kit (sigma, St. Louis, MO, USA) according to the manufacturer's recommendations. Briefly, cells were seeded and treated with the drugs for 24 h. Subsequently, the cells were lysed on ice with somatic cell ATP releasing reagent. Then the cell lysis to be assayed was mixed with solution including luciferase. Swirl briskly, transfer 0.1 mL to the reaction vial, and immediately measure the amount of light emitted with a luminometer (Charm Sciences, Malden, MA).

### Transfection experiment

A549 or H1975 cells were reverse transfected with either NF-κB overexpressing plasmid or vector using FuGene 6 (Roche Diagnostics) transfection reagent according to the manufacturer's instructions. Briefly, 2 μg plasmid DNA was diluted in Opti-MEM media and FuGene 6 transfection reagent was added in a ratio of 1:3 (w: v). The mixture was incubated at room temperature for 1 h in a six-well plate. Transfected cells were treated with DHA or 0.1% dimethylsulphoxide or for 24 h and then subjected to migration and invasion assay or western blot detection.

### *In vitro* wound-healing assay

Cells were seeded in six-well plates and allowed to grow overnight to confluence. The monolayer cells were scratched with a 200 μl pipette tip to create a wound and washed twice with serum-free DMEM to remove floating cells and the cells were then incubated in serum-free DMEM. The rate of wound closure was investigated by photography 24 h later. Each value was derived from three randomly selected fields.

### Boyden chamber assay

The migration and invasion assay was examined using 24-well Boyden chambers with 8 μm inserts coated without (migration) or with Matrigel (invasion) as previously described. 5 ×10^4^ cells were plated in the upper chambers without serum and supplemented with DHA and cultured at 37°C for 24 h. The cells that crossed the inserts were stained with crystal violet (Sigma) and then observed under phase-contrast microscopy and counted.

### Western blot analysis

Cells were lysed on ice with RIPA buffer. The protein concentration was determined by Bradford dye method. Equal amounts (20 to 40 μg) of cell extract were subjected to SDS-PAGE and transferred to PVDF membranes (Millipore) for antibody blotting. The membranes were blocked and incubated with primary antibodies and subsequently HRP-conjugated secondary antibody. Finally, the membranes were visualized using Dura Super Signal Substrate (Pierce) according to the manufacturer's instructions.

### Luciferase assay

Cells were plated in 48-well plates and incubated at 37°C to reach 70-80% confluence. The cells were washed with PBS and incubated with serum-free RPMI1640 without antibiotics for 6 h. 24 h after transfection, the cells were treated with DHA for additional 24 h and luciferase activity was measured using Dual Glo Luciferase kit (Promega) with Varioskan Flash multimode reader (Thermo Scientific). The Firefly luciferase activity was normalized to that of Renilla.

### Animal experiment

Female BALB/c athymic nude mice, 5- to 6-week-old, were pursued from the Experimental Animal Center of Xiamen University (China). All animals were fed with a standard diet *ad libitum* and housed in a temperature-controlled animal facility with a 12/12 hours’ light/dark cycle. All procedures were performed according to the NIH Guide for Care and Use of Laboratory Animals and were approved by the Bioethics Committee of Xiamen University. For experimental metastasis model, A549 cells (1×10^6^ cells) in 300μL PBS were injected directly into the tail veins of mice (28). One week after cell injection, the mice were randomized into a control group C0 (0 g/kg/d), or intervention groups C1 (50 mg/kg/d), or C2 (100 mg/kg/d) with stepwise increases in DHA doses. Each experimental group contained 5 mice. Mice were sacrificed after daily treatment for 28 days, and their lungs were weighed and subjected to tissue sectioning. To examine the metastases, 100 sequential sections (5 μm) were cut from the lungs of each mouse, and every 10th section was stained with hematoxylin and eosin (H&E).

### Analysis of the levels of Myc-GLUT1 on the plasma membrane

Cells were transduced with pcDNA3.2-Myc-GLUT1 vectors which express the GLUT1 with Myc tag in the first exofacial loop. At 48 h after transduction, the levels of Myc-GLUT1 on the cell surface and in whole cells were measured by IF staining in a flow cytometer as described [63]. The relative levels of Myc-GLUT1 on the cell surface were calculated after normalization with the total levels of Myc-GLUT1 in cells.

### Statistics

All *in vitro* experiments were performed three times and were presented as mean ± SEM. Statistical analysis was analyzed using the Statistical Package for Social Sciences (SPSS) software (version 16.0). *P* values were calculated using student's *t* test with a value of < 0.05 considered as statistically significant.

## SUPPLEMENTARY FIGURES


