# Supplementary material for: Repurposing the anti-malarial drug dihydroartemisinin suppresses metastasis of non-small-cell lung cancer via inhibiting NF-κB/GLUT1 axis
Source: Oncotarget. 2016 Nov 24;7(52):87271–83. doi: 10.18632/oncotarget.13536 (PMC5349987; doi:10.18632/oncotarget.13536)
Supplement: Supplementary file 1 [file oncotarget-07-87271-s001.pdf]

Repurposing the anti-malarial drug dihydroartemisinin suppresses metastasis of non-small-cell lung cancer via inhibiting NF-κB/GLUT1 axis

SUPPLEMENTARY FIGURES

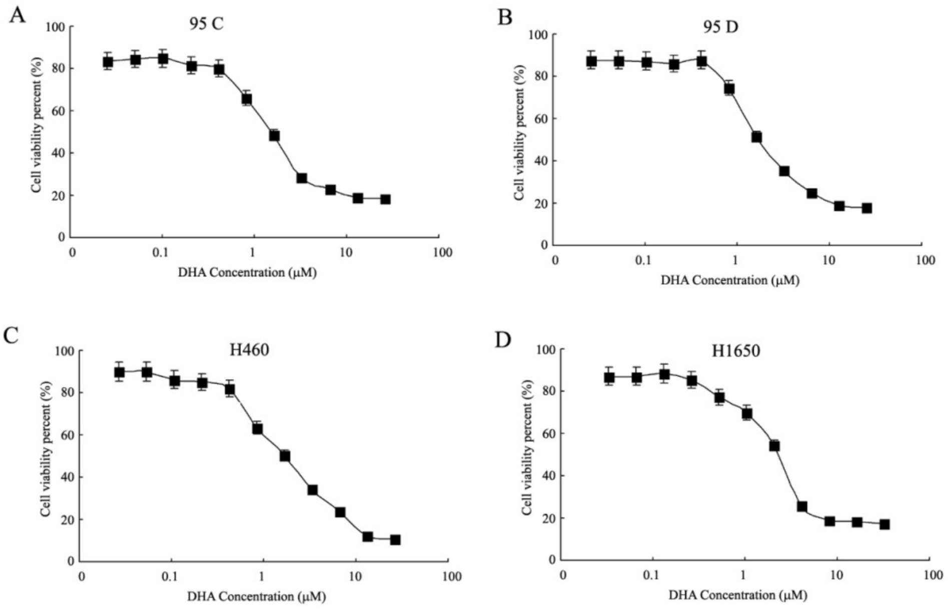

Supplementary Figure S1: DHA inhibits NSCLC cell proliferation. 95C, 95D, H460, and H1650 cells were treated with different concentration of DHA for 72 hours in DMEM containing 10% FBS. Cell survival was determined by MTT assays as described in Materials and Methods. Data are the mean ± SD of at least three independent experiments performed in triplicate.

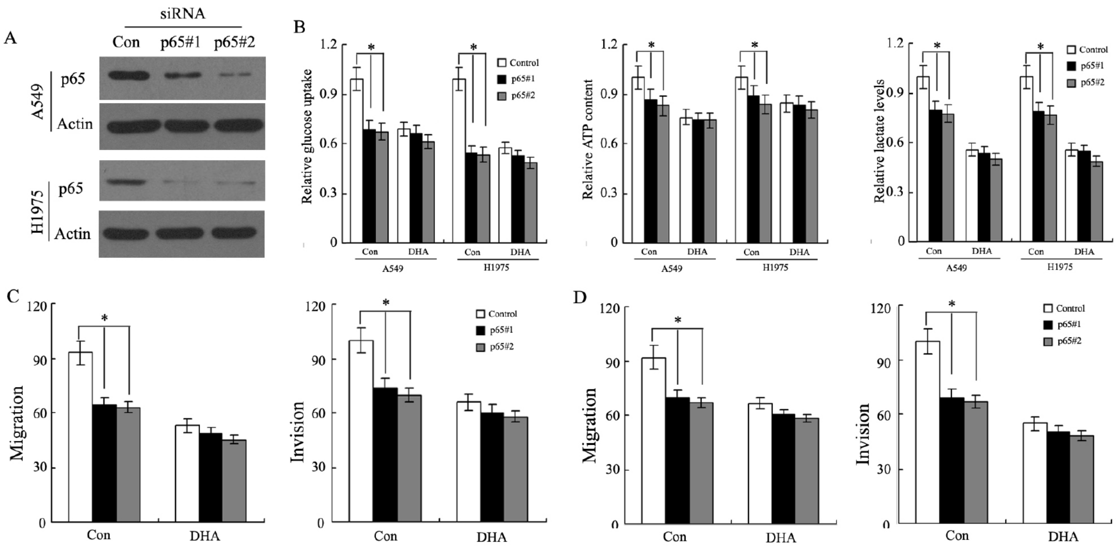

Supplementary Figure S2: DHA represses the NSCLC migration and invasion via the NF-κB pathway in vitro. A. Western-blot analysis of knockdown of p65 by siRNA in cells. **B.** DHA has no effect on glucose uptake, cell ATP content and lactate production in A549 and H1975 cells knockdown of p65. **C.** DHA has no effect on the invasion and migration abilities of NSCLC cells knockdown of p65. The indicated cells transfected with or without p65-siRNA (as indicated) for 24 hours were treated with or without DHA (15μM) for 24 hours, and then the migration and invasion assays (as indicated) were subsequently performed as described in Materials and Methods; n= 3; **, $P < 0.01$. A two-tailed Student t test was used for statistical analysis.

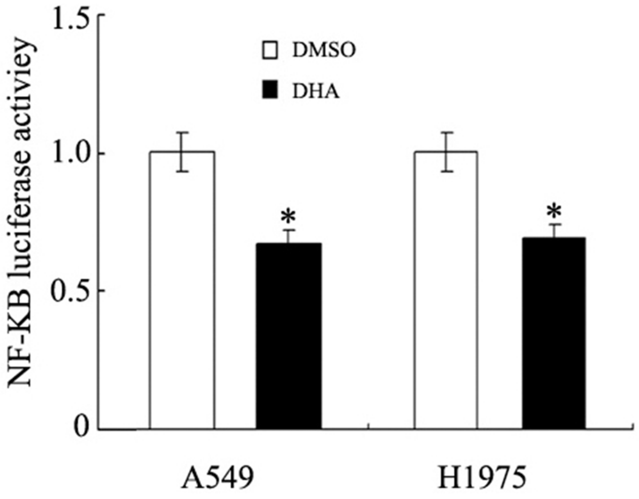

Supplementary Figure S3: DHA inhibits the luciferase activity of NF-κB. The indicated cells were transfected with a luciferase reporter for NF-κB, treated with or without DHA (15 μM) for 48 hours, and subjected to luciferase assays as described in Materials and Methods, $P < 0.05$. A two-tailed Student t test was used for statistical analysis.

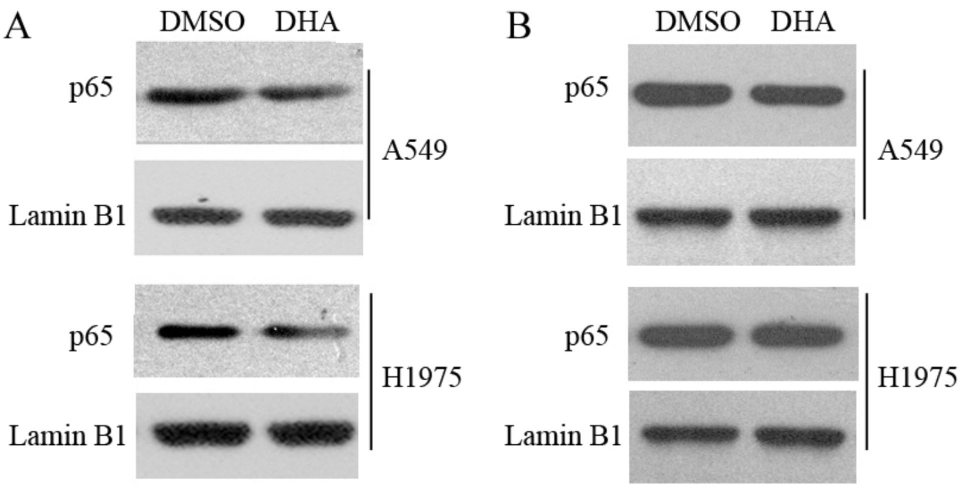

Supplementary Figure S4: Upregulated mTOR activation increased p65 nuclear translocation inhibited by DHA. A549 and H1975 cells were transfected with control **A.** and Rheb vector for 24h respectively, and then the indicated cells were treated with or without DHA (15 µM) for 48 hours and then subjected to cell fractionation and analyzed by Western blotting.
